# Salivary Biomarkers in Systemic Sclerosis Disease

**DOI:** 10.1155/2018/3921247

**Published:** 2018-03-12

**Authors:** Zeineb Zian, Joaira Bakkach, Amina Barakat, Naima Ghailani Nourouti, Mohcine Bennani Mechita

**Affiliations:** Biomedical Genomics and Oncogenetics Research Laboratory, Faculty of Sciences and Techniques of Tangier, Abdelmalek Essaâdi University, Tetouan, Morocco

## Abstract

Scleroderma or systemic sclerosis (SSc) is frequently detected at an advanced stage due to diagnosis difficulties. Salivary biomarkers, if existing, could be used for predictive diagnosis of this disease. Human saliva contains a large number of proteins that can be used for diagnosis and are of great potential in clinical research. The use of proteomic analysis to characterize whole saliva (WS) in SSc has gained an increasing attention in the last years and the identification of salivary proteins specific for SSc could lead to early diagnosis or new therapeutic targets. This review will present an overview about the use of WS in SSc studies. The proteomic technologies currently used for global identification of salivary proteins in SSc, as well as the advantages and limitations for the use of WS as a diagnostic tool, will be presented.

## 1. Introduction

Scleroderma or systemic sclerosis (SSc) is a rare systemic autoimmune disease affecting the connective tissue and characterized by involvement of the skin, blood vessels, and visceral organs. It is associated with dysfunction of the immune cells, fibroblasts, and endothelial cells. The etiology of SSc remains unknown [1]. SSc affects preferentially women, more often during and after their childbearing age [2]. Sex ratio varies in published series from 3 to 9 women for a man [3].

There are two main clinical forms of SSc that differ primarily in their degree of skin involvement: limited cutaneous scleroderma (lcSSc) and diffuse cutaneous scleroderma (dcSSc), which are associated with different clinical complications [4]. Oral manifestations are frequent in SSc [5], and the majority of oral clinical features start with tongue rigidity and facial skin hardening [6]. On the other hand, it was shown that SSc affects salivary glands [7], and these latter can also be subject to fibrosis in SSc patients [8].

The medications and fibrotic changes in salivary glands of patients with SSc can contribute to reduced salivary flow in these patients [9]. This diminishing of saliva production in SSc patients is mostly related to concomitant Sjögren's syndrome [10]. Although a little knowledge about salivary gland involvement in SSc has been reported in the literature, it was demonstrated, in a previous study, that salivary gland changes (increased expression of E-selectin and TNF-*α* and infiltration by mast cells) are detectable in the early stages of the disease, before the onset of skin changes and when the criteria for a diagnosis of SSc are absent [11].

The identification of salivary protein profiles could lead to early diagnosis or new therapeutic targets of SSc [12]. Furthermore, the presence of the biomarker may correlate with different clinical symptoms of the disease due to its absence in healthy subjects [13]. In the last years and with technological and analytical development, saliva has attracted an increased interest for use in diseases diagnosis and treatment. To date, there have been few reports aimed to use the WS in SSc research [7, 12–14]. Giusti et al. [14] performed, for the first time, a study in an attempt to characterize the WS protein profiles of patients with SSc using a proteomic research approach.

In this review we will give an overview of the use of WS in SSc research. The proteomic technologies currently used for global identification of salivary proteins in SSc, as well as the advantages and limitations for the use of WS in the disease, will be presented.

## 2. Whole Saliva

A number of proteomics researches contributed to the clarification and knowledge of the salivary proteome, and more than 2000 proteins and peptides have been found in WS and individual salivary glands [15]. Saliva is a mucoserous exocrine fluid produced by three major salivary glands (parotid, submandibular (SM), and sublingual (SL)) and other minor glands located under the oral mucosa [16]. Besides, WS comes also from local and systemic sources [17]. It is a combination of the secretions from the major and minor salivary glands, oral mucosa transudate, the gingival fold, desquamated epithelial and blood cells, nasal secretions, viruses, fungi, bacteria, and food debris [18]. WS contains hormones, immunoglobulins, proteins, enzymes, and mucosal glycoproteins [18]. It also contains a number of antimicrobial proteins which play an important role in reducing oral infections [19]. Its role in protecting oral structures has been well reported. Although saliva includes blood derived products, differently to serum, this oral fluid contains many locally secreted proteins which may be specific markers for some local diseases [16]. The presence in WS of many molecules that are circulating in the blood presents several advantages for disease diagnosis and prognosis as the collection of this fluid is noninvasive, safe, and easy; relatively low amounts of sample are needed and storage and transportation are not complicated [20, 21]. That is why, nowadays, WS is used as a diagnostic tool in clinical diagnosis, monitoring disease progression and management of patients [22, 23].

Passive drool saliva collection method is considered as the gold standard, but there are other saliva collection devices such as Salivettes [24, 25] which present a less viscosity and allow an easy handling as well as a better sample processing, particularly in some special cases such Xerostomia [26]. Unlike blood, due to its noncoagulating nature, saliva is easier to handle for diagnostic analysis procedures. The noninvasive collection procedures for saliva contribute to the procurement of repeated samples to follow the patients over time. Various collection and storage protocols of WS were described in published studies [7, 12–14, 27–36]. Due to the presence of circadian rhythms in WS flow rate and composition, WS collection should be made under standard conditions [37]. The variations in collection and/or storage procedures can change the salivary proteomic profiles after collection and therefore alter the biomarkers content and their detection [21], from where the need for adopting standardized procedures in saliva analysis. In fact, in order to avoid protein degradation, some authors have added 0.2% trifluoroacetic acid (TFA) to saliva sample [38]. Others have used a protease inhibitor cocktail and have stored the saliva at −20°C [39]. Furthermore, minimizing the time between collection and analysis of the sample has been proposed by some groups research [12–14, 36, 40]. Thus, it has been indicated that instead of using chemical inhibitors of proteolysis, collection of saliva into ice cold tubes could minimize proteolysis, as well as storage of the samples at −80°C rather than −20°C [41], in particular for storage at longer durations [42].

## 3. Applications of Whole Saliva for Systemic Sclerosis Study

In the recent years, proteomic approaches started to be used in the study of WS from patients with SSc. Knaś et al. [7] study was the first to describe the alteration on the salivary glands function in both subsets of SSc.

Among the different proteomic approaches, saliva has been studied using several techniques, either separately, or, more often, in combination such as one-dimensional polyacrylamide gel electrophoresis (1D-PAGE), two-dimensional polyacrylamide gel electrophoresis/mass spectrometry (2-DE/MS), capillary electrophoresis (CE), 2D-liquid chromatography/mass spectrometry (2D-LC/MS), nuclear magnetic resonance (NMR), matrix-assisted laser desorption ionization-time of flight mass spectrometry (MALDI-TOF/MS), surface enhanced laser desorption/ionization time-of-flight mass spectrometry (SELDI-TOF/MS), Western blot, electrospray ionization (ESI), immunoassays (radio-immune assays, immunoradiometric assays, enzyme immuno-assays, and enzyme-linked immunosorbent assays) and lectin binding assays on PAGE gels [15, 22, 43–54]. The choice for the technique is depending on the objectives of the study and on the salivary proteins of interest. It has been reported that 2-DE combined with mass spectrometry has been widely used to study salivary proteins [14, 15, 49]. However, due to its limitations it is not the most important tool used in this field and does not allow the study of the complete proteome. In fact, other techniques have been shown to be more performant, including a variety of chromatographic combinations that has successfully characterized more than 3000 different components in saliva [38, 55–57]. Surface chromatography combined with MALDI-TOF/MS has allowed rapid and high-throughput detection of important proteins and peptides [58]. From our knowledge, only four studies focused on WS in SSc have been published, and only one study [14] has identified the salivary biomarkers in these patients. In these proteins separation was achieved by using 2-DE technique with subsequent protein identification being made by MALDI-TOF-MS (Table 1).

## 4. Salivary Proteins in Systemic Sclerosis

To date, there is a lack of early diagnostic markers, and the time between the diagnosis and symptom onset can be translated by years. Identification of the salivary proteins biomarkers involved in SSc may contribute to the early detection of the disease. The specific salivary markers identified so far were reported by Giusti et al. [14] in a study including 15 patients with dcSSc, in which they compared the differences between WS of SSc patients and control subjects. Indeed, it was reported that both previously identified and newly identified proteins occurred in WS of SSc patients but did not match with healthy subjects. Some of these proteins, such as keratin 6L, psoriasin, TPI, and Arp2/3 complex, might play a pathological role in SSc, suggesting that some of them may be considered as new therapeutic targets or diagnostic markers for SSc. It was found also that, except Keratin 6L, the expression of most of the called typical salivary proteins like *α*-amylase, prolactin-inducible protein precursor, albumin, or cystatins remained unchanged between control subjects and patients. In contrast, the same research team showed that the expression of these normal proteins was altered in Sjögren's syndrome patients compared to the controls, with decreased levels of some salivary proteins [36] (Table 2).

Among those proteins, three that belong to the S100 calcium- and zinc-binding protein family related to inflammation have been identified: calgranulin A (S100A8), calgranulin B (S100A9), and psoriasin (S100A7) [12, 14, 45, 59]. S100A8 and S100A9 are mainly localized in the cytosol of neutrophils and are involved in the metabolism of arachidonic acid in human neutrophils [43, 60]. Some findings suggest that high concentrations of S100A8 and S100A9 might play a role in inhibiting the matrix metalloproteinases activity by the sequestration of zinc [59, 61]. This inhibition or reduced activity of MMP plays a crucial role in reducing extracellular matrix degradation in SSc individuals and leads to extensive fibrosis of this disease. Regarding psoriasin (S100A7), it was firstly identified by Madsen et al. [62], as a protein expressed in epithelial cells of the psoriatic skin. Increase of psoriasin expression has been also observed in WS of patients with dcSSc [14]. Although the biological effect of psoriasin in SSc remains unknown, a significant association of this protein and pulmonary involvement of dcSSc has been demonstrated [13]. Arp2/3 complex has been newly identified in WS [14]. This complex plays a role in the regulation of actin polymerization in cells, and it is necessary for neutrophil chemotaxis and phagocytosis [63]. The ß2-microglobulin is a component of MHC class I molecules which may play a role in the immune dysregulation of SSc [14]. Triose phosphate isomerase (TPI) and glyceraldehyde-3 P-dehydrogenase (GAPDH) 190 are glycolytic enzymes present in cytoplasm that may act as autoantigens in SSc and also in other autoimmune diseases such as systemic lupus erythematosus [64]. Regarding cyclophilin A, its contribution to the pathogenesis of immune-mediated endothelial activation and dysfunction was suggested by Kim et al. [65]. It is involved in the expression, folding, and degradation of proteins and catalyzes the cis-trans isomerization of proline imidic peptide bonds in oligopeptides [66]. Cystatin B is an intracellular thiol proteinase inhibiting [14, 44, 67]. However, its role in SSc has not been reported so far.

## 5. Advantages and Limitations

In the recent years and with the advances in proteomic technologies, salivary research emerged as an important area for the diagnosis of several local and systemic diseases. As mentioned above, saliva showed several advantages for systemic diseases research as well as for SSc including mainly safety and easy collection using simple, inexpensive, and noninvasive methods. The presence of several serum components in saliva has great benefits for research of new biomarkers. Moreover, due to its noncoagulating nature, saliva is easier to handle in diagnostic analysis procedures [15, 20, 22, 23]. Our knowledge about specific advantages and limitations of the use of this tool with diagnosis purposes in SSc is still limited. We mention in this review a raised concern for the use of WS in SSc patients with Sjogren's syndrome. These latter were shown to have generally a reduced salivary flow rate that could limit the collection and use of WS as research material for this group [7], but this needs to be more investigated. The most known advantages and limitations of WS that are likely to be extrapolated for SSc are presented in Table 3.

## 6. Conclusion

In conclusion, salivary biomarkers study is becoming an important part of the diagnosis of several diseases. Identification of salivary proteins in SSc is a promising finding that paved the way to new diagnostic biomarkers for this pathology, but this needs to be more investigated as there are so far only few studies published in this regard.

Several approaches (SELDI-TOF/MS, HPLC, and other affinity chromatography techniques) hold promise for salivary proteomic analysis to discover and validate new biomarkers or therapeutic targets.

Deeper and more comprehensive studies are required to elucidate the functional significance of these proteins during the SSc and to improve diagnosis as well as treatment. In addition, larger populations are needed to validate and generalize these results in future studies of salivary proteomic. On the other hand, research to the salivary proteome in SSc patients from different regions of the world is required due to the variability in genetic and environmental factors of each subject.

## Figures and Tables

**Table 1:** Summary of published studies using the WS in SSc.

Study	Saliva sample	Patients/controls	Analytical methods	Findings
Giusti et al. [14]	UWS	15/15	2-DE, MALDI-TOF/MS	Presence of 9 proteins only in SSc (calgranulin A, calgranulin B, psoriasin, Arp2/3 complex, ß2-microglobulin, TPI, GAPDH, cyclophilin A, and cystatin B).

Baldini et al. [13]	UWS	44/80	SDS/PAGE, Western blot	Significant association of psoriasin with pulmonary involvement in dcSSc.

Knaś et al. [7]	UWS/SWS	97/55	ELISA, Spectrophotometrically	(i) In UWS of dcSSc and lcSSc: (1) Salivary flow, the output of total protein, and peroxidase activity were significantly lower. (2) sIgA and lactoferrin were significantly higher. (ii) In SWS: (1) In lcSSc, the total lysozyme and peroxidase activity were significantly higher. (2) In dcSSc, the salivary flow was significantly lower and the total sIgA and peroxidase activity were significantly higher.

Giusti et al. [12]	UWS	134/74	ELISA	Significant correlation between salivary psoriasin and DLCO in SSc.

dcSSc, diffuse cutaneous systemic sclerosis; DLCO, diffusion capacity of carbon monoxide; ELISA, enzyme-linked immunosorbent assay; lcSSc, limited cutaneous systemic sclerosis; MALDI-TOF, matrix-assisted laser desorption ionization; MS, mass spectroscopy; SDS/PAGE, sodium dodecyl sulfate polyacrylamide gel electrophoresis; SSc, systemic sclerosis; SWS, stimulated whole saliva; UWS, unstimulated whole saliva; 2-DE, two-dimensional gel electrophoresis.

**Table 2:** Salivary proteins identified in WS of SSc patients according to Giusti et al. [14].

Proteins	Swiss-Prot/NCBI	Function
Calgranulin A	P05109	Present in chronic inflammation and in epithelial cells constitutively or induced during dermatoses. Involved in the metabolism of arachidonic acid in human neutrophils. Seem to have a major role in inflammatory and immunological responses. May interact with components of the intermediate filaments in monocytes and epithelial cells. May play a role in inhibiting the matrix metalloproteinases activity.

Calgranulin B	P06702	Present in acute and in chronic inflammation. Stimulate neutrophil adhesion. Involved in the metabolism of arachidonic acid in human neutrophils. May play a role in inhibiting the protein kinases and the matrix metalloproteinases activity. May interact with components of the intermediate filaments in monocytes and epithelial cells.

Cystatin B	P04080	Proteinase inhibiting properties. Tightly binding reversible inhibitor of cathepsins L, H, and B.

Psoriasin	P31151	Present in fetal ear, skin, and tongue and human cell lines. Highly expressed in psoriasis and in other inflammatory skin diseases. Seem to participate in tumor progression. Also highly expressed in the urine of patients with bladder squamous cell carcinoma.

ß2-Microglobulin	Q6IAT8	Component of the MHC class I molecules.

Cyclophilin A	P62937	Involved in expression, folding, and degradation of proteins. Catalyze the cis-trans isomerization of proline imidic peptide bonds in oligopeptides.

Glyceraldeyde-3 P-dehydrogenase	P04406	Glycolytic enzymes present in the cytoplasm. Play a role in degradation of carbohydrate and glycolysis.

Triose phosphate isomerase	P60174	A highly conserved glycolytic enzyme. Mediated by glycolysis in red blood cells and in brain cells. Biosynthesis of carbohydrate and gluconeogenesis.

Actin-related protein 2/3 complex subunit 2	O15144	Strong candidate for the control of actin polymerization in chemotaxis.

**Table 3:** Advantages and limitations of WS as a diagnostic tool.

Advantages	Limitations
(i) Readily accessible and informative biofluid.	(i) Many informative molecules in lower amounts of saliva.
(ii) Easy, safe, inexpensive, and noninvasive diagnostic approach.	(ii) Centrifugation may also remove other proteins.
(iii) Noncoagulating nature.	(iii) Presence of several proteases degrading protein biomarkers.
(iv) More sensitive and more specific markers for oral diseases.	(iv) Difficult to have saliva completely free of stimulation which influences the results.
(v) Simple collection and minimal equipment required.	(v) Possibility of assaying proteins only after recent Exposure.
(vi) Storage and transportation at low cost.	(vi) Difficult detection with low concentrations of proteins of interest in saliva.
(vii) Less amounts of sample	
(viii) Contains serum constituents	
